# Transcriptional regulators dictate innate lymphoid cell fates

**DOI:** 10.1007/s13238-017-0369-7

**Published:** 2017-01-20

**Authors:** Chao Zhong, Jinfang Zhu

**Affiliations:** 1 Molecular and Cellular Immunoregulation Unit, Laboratory of Immunology, National Institute of Allergy and Infectious Diseases, National Institutes of Health, Bethesda, MD 20892 USA; 2 Institute of Systems Biomedicine, School of Basic Medical Sciences, Peking University Health Science Center, Beijing, 100191 China

**Keywords:** innate lymphoid cell, transcription factors, GATA-3, Id2

## Abstract

Research on innate lymphoid cells (ILC) has recently been a fast paced topic of immunological research. As ILCs are able to produce signature Th cytokine, ILCs have garnered considerable attention and have been described to represent the innate counterpart of the CD4^+^ T helper (Th) cells. The development and function of ILCs are precisely regulated by a network of crucial transcription factors, which are also involved in the development or differentiation of conventional natural killer (cNK) cells and T cells. In this review, we will summarize the key transcriptional regulators and their functions through each phases of ILC development. With the phase of ILC lineage commitment, we will focus in particular on the roles of the transcription regulators Id2 and GATA-3, which in collaboration with other transcriptional factors, are critically involved in the generation of ILC fate determined progenitors. Once an ILC lineage has been established, several other transcription factors are required for the specification and functional regulation of distinct mature ILC subsets. Thus, a comprehensive understanding of the interactions and regulatory mechanisms mediated by these transcription factors will help us to further understand how ILCs exert their helper-like functions and bridge the innate and adaptive immunity.

## INTRODUCTION

Research on a new population of lymphocytes, innate lymphoid cells (ILCs), has been on the rise during the past few years. Compared to other cells in the innate immune system, ILCs are unique in that they may produce and secrete cytokines that were classically regarded as CD4^+^ Th cell products (Eberl et al., 2015b). Thus, ILCs are considered to be an important component of the innate immune system and their development and functionality has drawn considerable attention in the field. Innate immunity is an evolutionarily ancient system. A major feature of innate immunity is the antigen non-specificity. Pattern recongnition (PR) is a well-known manner to initiate an innate response. The innate immune system recognizes pathogen-associated or damage-associated molecular patterns (PAMPs or DAMPs) through pattern recognition receptors (PRRs) in a semi-specific manner (Brubaker et al., 2015). Signals transduced downstream of PRRs may promote the production of pro-inflammatory cytokines or chemokines, including IL-1β, TNFα, IFN-β, IL-8, etc.

In contrast, the adaptive immune system has evolved to generate exquisitely specific responses to particular antigens, which eventually leads to immunological memory. CD4^+^ T cells are one of the key players in the adaptive immune system. Early T cell precursors (ETPs) develop through multiple selection steps, including CD4^−^CD8^−^ double negative (DN), CD4^+^CD8^+^ double positive (DP), and CD4^+^ or CD8^+^ single positive (SP) in the thymus. Once the T cell precursors have experienced β-selection, positive and negative selection, they eventually become *bona fide* naïve CD4^+^ or CD8^+^ T cells (Germain, 2002). Several crucial transcription factors are involved in regulating and orchestrating this process, including TCF1, TOX, Bcl11b, GATA-3, Th-Pok, and Runx3, etc. (Yui and Rothenberg, 2014). Naïve CD4^+^ T cells, after migrating out of the thymus to the periphery, will further differentiate into distinct effector cells upon encountering antigen-laden antigen presenting cells. During this process, the signals triggered by TCR, co-stimulatory receptors and cytokine receptors influence the ultimate effector T helper cell fate of the naïve T cell (O’Shea and Paul, 2010). For example, IL-12 drives the differentiation of type 1 T helper (Th1) cells; IL-4 promotes type 2 T helper (Th2) cells; and IL-6 together with TGF-β facilitates the generation of IL-17-producing T helper (Th17) cells. Differentiated Th effectors are capable of expressing their signature effector cytokines—IFN-γ for Th1, IL-4 for Th2, and IL-17 for Th17 cells. The transcription factors that are deterministic for the differentiation and functions of Th cell subsets, are referred to as master transcription factors and include T-bet for Th1, GATA-3 for Th2, and RORγt for Th17 cells. The precise functions of these master lineage regulators during CD4^+^ T cell activation and Th effector differentiation have been extensively studied using gain or loss of function animal models.

The production of signature effector cytokines had historically been considered a unique feature of CD4^+^ Th cells in the adaptive immune system, until the discovery of ILC populations. These innate lymphocytes were overlooked possibly due to their lack of expression of any known lineage markers and their enrichment mainly in the non-lymphoid tissues. The first descriptions of a non-T non-B lymphocyte population that produced the Th2 cytokines, IL-5 and IL-13, began the innate lymphoid cell field (Fallon et al., 2006; Moro et al., 2010; Neill et al., 2010; Price et al., 2010). It is now well known that there are several distinct ILC subsets that can express signature cytokines like Th cells (Eberl et al., 2015a). For example, group 2 ILCs (ILC2s) can produce the effector cytokine IL-5 and IL-13 like Th2 cells, group 3 ILCs (ILC3s) can produce IL-22, IL-17a, and IL-17f as Th17/Th22 cells, and group 1 ILCs (ILC1s) can produce IFN-γ and TNF-α like Th1 cells. Interestingly, in addition to their mirrored cytokine repertoire, both CD4^+^ T cells and ILC subsets also utilize a similar set of transcriptional factors for their development, differentiation and functions (Artis and Spits, 2015; Zhong and Zhu, 2015b; Zook and Kee, 2016).

In addition to their ability to produce signature cytokines, ILCs are interesting in that they are primarily tissue resident lymphocytes. ILC progenitors are developed in the bone marrow, while mature ILCs are mainly enriched in peripheral tissues such as gastrointestinal (GI) tract, lung, liver, and skin. Recent studies from parabiosis experiments have confirmed that the vast majority of ILCs are tissue-resident (Gasteiger et al., 2015). In addition, a few reports have addressed the question of how bone marrow ILC progenitors home to peripheral tissues. For example, ILC2s gain the gut homing receptor CCR9 and Integrin α_4_β_7_ during their development in bone marrow, and thus ILC2s directly migrate to and reside in the gut after maturation. On the other hand, the precursors of ILC1 and ILC3s may initially express the homing receptor CCR7, which directs them to lymphoid organs such as the spleen and lymph nodes. Then upon encountering retinoic acid, ILC1 and ILC3 cells may down-regulate CCR7 and up-regulate both CCR9 and Integrin α_4_β_7_, which eventually guides these cells to the gut (Kim et al., 2015). However aside from the gut, it is currently unclear how ILCs may migrate to other peripheral tissues. The process of ILC colonization is further complicated by the fact that ILC precursors are also found in periperal tissues (Bando et al., 2015). At the fetal stage, some ILC progenitor cells have been observed to migrate to and reside in the proximal gut, where they are responsible for the generation of Peyer’s patches. Interestingly, these precursors have the capacity to develop into all three ILC subsets and may thus serve as a resident source of ILCs.

Functionally, ILCs are crucially important in providing protection against pathogens in the early stages of an immune response. For example, ILC2s are an important source of IL-5, IL-13, and IL-9 after an infectious challenge with helminth pathogens (Fallon et al., 2006; Neill et al., 2010; Price et al., 2010; Wilhelm et al., 2011). The production of these cytokines is required for the efficient recruitment of eosinophils (Nussbaum et al., 2013). ILC2s also participate in wound healing via secretion of amphiregulin (Monticelli et al., 2011). Additionally, ILC2-derived cytokines may promote the beigeing of adipose tissue and may thus affect adipose tissue homeostasis (Lee et al., 2015). ILC3 cells are RORγt-expression ILCs. A special subset of ILC3s is the LTi cell which is required for the formation of secondary lymphoid structures such as lymph nodes and Peyer’s patches (Finke, 2005). ILC3s are enriched in the GI tract and are associated with inflammatory bowl diseases. ILC3s are the main source of IL-22 in homeostatic conditions and in the early stages of certain inflammatory responses (Cella et al., 2009; Luci et al., 2009; Sanos et al., 2009; Takatori et al., 2009). IL-22 acts on intestinal epithelium to regulate the homeostatic self-renewal of epithelial stem cells (Hanash et al., 2012; Lindemans et al., 2015) and the production of antimicrobial peptides (Dudakov et al., 2015). Additionally, ILC3-derived IL-22 and lymphotoxin are also required for the fucosylation of the gut epithelial cells by stimulating the expression of fucosyltransferase Fut2 (Goto et al., 2014; Pickard et al., 2014). The fucose residues on the surface of the gut epithelium may provide nutrients for the gut microbiota, thus maintaining gut homeostasis. Recent studies have also indicated that ILC3s in other peripheral tissues, including the skin (Pantelyushin et al., 2012), may also play crucial roles in maintaining the local microenvironment. ILC1s have similar features to conventional NK cells, aside from their lack of cytotoxicity. However, ILC1s are more efficient producers of IFN-γ and TNF-α during a type 1 response and ILC1s provide the innate protection against *Toxoplasma gondii* infection (Klose et al., 2014).

ILCs may directly or indirectly regulate the adaptive immune response. For example, a deficiency in ILC2s may result in a dramatic reduction in Th2 responses (Oliphant et al., 2014). ILC2-derived IL-13 may promote DC migration to the draining lymph node where the DCs can facilitate T cell activation and Th2 differentiation (Halim et al., 2014). IL-13 also licenses DC subsets to secrete the chemokine CCL17, which is required for the recruitment of memory CD4^+^ T cells (Halim et al., 2016). In addition, ILC2s may express the antigen-presenting molecule MHCII and can crosstalk directly with Th2 cells to augment immune responses (Oliphant et al., 2014). However, a recent report indicates that a normal Th2 response may occur in the absence of ILC2s (Van Dyken et al., 2016), arguing that the ILC2-derived IL-13 and the potential antigen-presenting functions of ILC2s may not be necessary to mount an effective Th2 response. Interestingly, ILC3s may also express MHCII, however ILC3-dependent antigen presentation represses commensal antigen specific CD4^+^ effector T cells, due to a lack of or low expression of co-stimulatory molecules on ILC3s (Hepworth et al., 2015; Hepworth et al., 2013).

Thus as the field continues to explore the functions of ILCs, new and novel functions of various tissue-resident ILC subsets are being reported. However the development and function of ILCs are, like all cells, subject to transcriptional regulation and control. Therefore in the remainder of this review, we will summarize the transcription factors that have been shown to critically regulate the functions and development of tissue resident ILCs.

## TRANSCRIPTIONAL REGULATION OF ILC DEVELOPMENT

**Figure 1:**
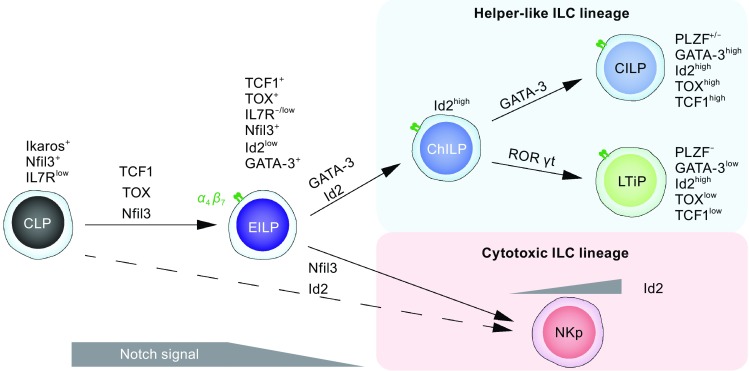
**Critical transcription factors regulate ILC development**. After the stage of common lymphoid progenitor (CLP), transcription factors such as TCF1, TOX and Nfil3, together with the Notch signals, promote the generation of an early innate lymphoid progenitor (EILP) expressing Integrin α_4_β_7_. EILP has the potential to become both the helper-like innate lymphoid cell (ILC) lineage and the cytotoxic conventional nature killer (cNK) lineage, but has lost the capacity to generate T cells, B cells or dendritic cells. GATA-3 is required for the development of common helper-liker innate lymphoid progenitor (ChILP), which expresses high levels of DNA binding inhibitor Id2. The development of the cytotoxic innate lineage does not require GATA-3, but require Id2 whose expression level is low at the progenitor stage and gradually increases during NK cell development. Within the ChILP population, some cells transiently express PLZF and are committed to non-LTi ILC lineages in a GATA-3-dependent manner. Progenitors that have not previously expressed PLZF generate the LTi cells in a RORγt-dependent manner

To fully understand the function of ILCs, we need first to understand how this lineage is generated. At present, the regulatory mechanisms of innate lymphoid cell development have been quite elusive. Our initial knowledge on the generation of these cells mainly stemmed from the phenotypes of global gene knockout mice. Nevertheless, it is now clear that the transcriptional regulators that are associated with cNK or T cell development are also involved in ILC development, suggesting that ILCs and T cells have a close developmental relationship. Research on the transcriptional regulation of ILC development became feasible once common ILC progenitor cells were identified. Below is a summary of the critical transcription factors that have been identified to be involved in ILC development (Fig. 1).

### Id2

Studies on ILC development have advanced progressively since the identification of the ILC fate determined progenitors, the common helper-like innate lymphocyte progenitors (ChILPs) (Klose et al., 2014). Id2 is expressed by ILCs and cNK cells and is required for their development (Verykokakis et al., 2014; Yokota et al., 1999). Id2 belongs to the “inhibitor of DNA binding protein” family, which is comprised of Id1-4. Id proteins share a highly conserved helix-loop-helix (HLH) domain and can negatively regulate other HLH domain-containing transcription factors by forming heterodimers with their HLH domain; thus inhibiting their ability to bind to DNA. E-box proteins, including E2A, E2-2, HEB, are one example of a subset of basic HLH transcription factors that are antagonized by Id proteins (Kee, 2009). Id2 is required for all innate lymphocytes development, partially due to their repression of the E-box proteins (Verykokakis et al., 2014). Indeed, an additional deficiency of E2A in *Id2* ^−/−^ mice rescues a defect in lymphoid tissue inducer (LTi) cell development as well as a defect in the formation of secondary lymphoid structures (Boos et al., 2007). ChILP cells are defined as lineage^−^IL-7R^+^Flt-3^−^Integrin α_4_β_7_^+^CD25^−^Id2^high^ progenitor cells that have committed their fates to an ILC lineage (Klose et al., 2014) (Fig. 1). Some transcription regulators associated with the up-regulation of Id2 have also been identified and will be discussed later. However, the precise mechanisms through which Id2 mediates ILC development and the environmental signals that induce Id2 expression in ChILP cells are still elusive.

### GATA-3

In addition to Id2, GATA-3 is another transcriptional regulator that is required for the development of all innate lymphocytes aside from cNK cells (Yagi et al., 2014). GATA-3 is well known for its involvement in T lymphocyte development at various stages (Ho et al., 2009). During CD4^+^ T cell development, GATA-3 needs to be up-regulated to a proper high level in order to induce Th-pok expression and thus to direct CD4^+^ T cell generation (Wang et al., 2008). Deficiency in GATA-3 at the CD4^+^CD8^+^ double positive (DP) stage results in a failure of CD4^+^ T lineage commitment (Pai et al., 2003), whereas hyperexpression of GATA-3 by a GATA-3 transgene is toxic to the cells (Taghon et al., 2007). Furthermore, overexpression of GATA-3 at the DN stage redirects these cells developing into mast cells (Taghon et al., 2007). Thus, the level of GATA-3 expression during T cell development needs to be carefully controlled. We have previously found that ILCs require GATA-3 for their development. A conditional *Gata3* deficiency starting from the hematopoietic stem cell stage mediated by *Vav*Cre results in a developmental defect affecting almost all ILCs (Yagi et al., 2014), consistent with another report showing that a *Gata3* germline deletion causes the failure of ILC3 development (Serafini et al., 2014). By tracing GATA-3 expression along ILC development, we and others have found that GATA-3 expression is undetectable at the common lymphoid progenitor (CLP) stage, and that CLP development in *Gata3* ^fl/fl^ *Vav*Cre mice seems to be normal. However within the ChILP cells, a subset expresses high GATA-3 levels, suggesting that high levels of GATA-3 expression at this progenitor stage may be required for the development of all ILCs. As GATA-3 is dispensable for cNK cell development but required for the optimal function of mature cNK cells (Samson et al., 2003), it is intriguing to explore whether the GATA-3 expression levels in innate lymphocyte progenitors can determine their lineage fates towards either helper-like ILCs or cytotoxic cNK cells, in a similar manner to the function of GATA-3 in CD4^+^ and CD8^+^ T cell lineage commitment.

## PLZF

In addition to ChILP cells, another specialized ILC progenitor population has been identified based on the expression of the transcription factor PLZF; namely the lineage^−^PLZF^+^ common innate lymphoid progenitor (CILP) (Constantinides et al., 2014) (Fig. 1). Further characterization of these cells has indicated that they are IL-7R^+/−^, Flt-3^−^, Integrin α_4_β_7_^+^, Id2^+^, and GATA-3^+^. Interestingly, PLZF is only transiently expressed at the ILC progenitor stage. Using a fate-mapping mouse strain to trace current and former PLZF-expressing ILC progenitor-derived cells or by transferring PLZF-expressing CILPs, Bendelac and his colleagues have demonstrated that the progenies of PLZF-expressing progenitors include all ILCs but not LTi cells, indicating that LTi cells develop from a distinct progenitor, which is separated from the PLZF-expressing progenitors that give rise to non-LTi ILCs. However, PLZF itself is not absolutely required for ILC development, despite a modest reduction of ILC2 cells in *Zbtb16* ^−*/*−^ mice (PLZF gene) (Constantinides et al., 2014).

### Notch

The Notch signaling pathway is highly conserved in most multicellular organisms and it plays critical roles during lymphoid lineage commitment. For example, T and B cell fate in the early stages after CLP is determined by the on and off status of Notch signals (Radtke et al., 2013). Notch signals are also critically involved in ILC development and functional regulation (Lee et al., 2011; Possot et al., 2011; Wong et al., 2012), possibly through their regulation of *Gata3* and/or *Tcf7* expression, which needs to be studied further. Interestingly, while Notch signals are required for LTi development, sustained Notch signals can block the generation of LTi cells (Chea et al., 2016). Therefore, Notch signaling seems to be dynamically regulated and its functions may be stage specific during ILC development.

### Nfil3

Nuclear factor interleukin-3 (Nfil3, also known as E4BP4) has been recently reported to be a critical transcription factor for the development of all ILCs (Seillet et al., 2014; Xu et al., 2015; Yu et al., 2014), and the development of cNK cells (Gascoyne et al., 2009). Nfil3 can be induced by IL-7 signaling in CLPs (Xu et al., 2015). For both ILC and cNK cell development, Nfil3 can efficiently activate Id2 expression, probably explained by its direct binding to the *Id2* locus (Xu et al., 2015). However, Nfil3 is not indispensible for Id2 expression in innate lymphocyte progenitors since both cNK cells and ILCs can still develop in the *Nfil3* ^−/−^ mice, albeit at lower numbers.

### TOX

Thymocyte selection-associated HMG box protein (TOX) belongs to another evolutionarily conserved high mobility group (HMG)-box family. TOX is known to be essential for the development of both adaptive T cells and innate cNK cells. TOX is transiently expressed during the β-selection and positive selection of T cell development and may be involved in the transition from CD4^low^CD8^low^ to CD4^+^CD8^low^ (Aliahmad and Kaye, 2008). TOX is also expressed in developing and mature cNK cells (Aliahmad et al., 2010). Nfil3 may bind to the *Tox* locus and may positively regulate its expression (Yu et al., 2014). Tox deficiency results in a blockage of cNK development at the lineage^−^IL15Rα^+^NK1.1^−^DX5^−^ progenitor stage, but the blockage is not associated with Id2 expression (Aliahmad et al., 2010). *Tox* ^−/−^ mice have a profound defect in LTi generation, as indicated by the lack of peripheral lymph nodes and a significantly reduced Peyer’s patch frequency and size (Aliahmad et al., 2010). With regard to ILC development, a *Tox* deficiency results in a dramatic reduction of ChILPs (Seehus et al., 2015). A comparison of gene expression in wild type and *Tox* ^−/−^ ChILPs suggests that a decrease in Notch signals, in concert with their downstream target genes, including *Tcf7*, *Hes1*, *Gata3*, and *Bcl11b* might be responsible for the developmental defect in ChILPs.

### TCF1

T-cell factor 1 (TCF1, encoded by *Tcf7*) is another member of the HMG box-containing transcription factors. It is also involved in the development of both T cells and ILCs. TCF1 level during T cell development is up-regulated as early as the early T cell progenitor (ETP) stage, and together with its close homolog, lymphoid enhancer binding factor 1 (LEF-1), TCF1 promotes T cell development possibly by acting as a downstream effector of Notch signaling (Steinke et al., 2014; Weber et al., 2011). It has been recently reported that TCF1 is critical for the development of multiple ILC subsets including ILC2s and ILC3s (Mielke et al., 2013a; Yang et al., 2013). TCF1 expression is induced before the ChILP stage. Based on TCF1 expression, an earlier innate lymphocyte progenitor, termed EILP, has been identified (Yang et al., 2015) (Fig. 1). The EILP has the potential to develop into either cNK cells or ILCs, but has lost its capacity to become T or B cells. EILPs, which are different from ChILPs, express very low levels of Id2, but substantial levels of Nfil3 and TOX transcripts. Thus Nfil3 and/or TOX may be responsible for inducing TCF1 expression, and thus the generation of EILPs. Therefore, Nfil3, TOX, and TCF1 may represent a few upstream transcription factors that are required to set up the environment for Id2 up-regulation and thus ChILP generation. Besides inducing Id2, TCF1 may up-regulate GATA-3 expression, presumably through similar GATA-3 regulation mechanisms found during ILC2 development.

Although the above transcription factors are essential for the generation of ILCs, the regulatory network among them in ILC progenitors, and the precise mechanism through which ILCs are developed still need further investigation. Our current knowledge about ILC development is still quite limited. As ILC progenitors are rare populations in the bone marrow, their low frequency makes it very difficult to establish the precise regulatory mechanisms within these cells. The regulation of ILC development is likely to be far more complicated than we have discussed. Additional un-described transcriptional regulators or environment cues may play large roles in governing progenitor cell fates during development. With the development of more advanced technologies, including single cell transcriptomic analyses, it is now possible to profile the whole transcriptomes of rare progenitor populations and to reveal their heterogeneity (Bjorklund et al., 2016; Gury-BenAri et al., 2016; Ishizuka et al., 2016; Yu et al., 2016). Further studies of ILC progenitors are ultimately necessary to further understand the evolution and lineage specification of the lymphoid system in mice and clinical patients.

## SPECIFIC REGULATION OF ILC SUBSETS

In addition to the aforementioned transcription factors that affect the development of ILC progenitors, additional transcription factors play a role in the formation of distinct mature ILC subset. These transcription factors are mostly expressed in or after the ChILP stage. However, some of the transcription factors mentioned above are also involved in the maintenance or functional regulation of select ILC subsets. Hereafter we discuss the transcriptional regulators that function in each particular ILC subsets.

### ILC2-associated transcription factors

**Figure 2:**
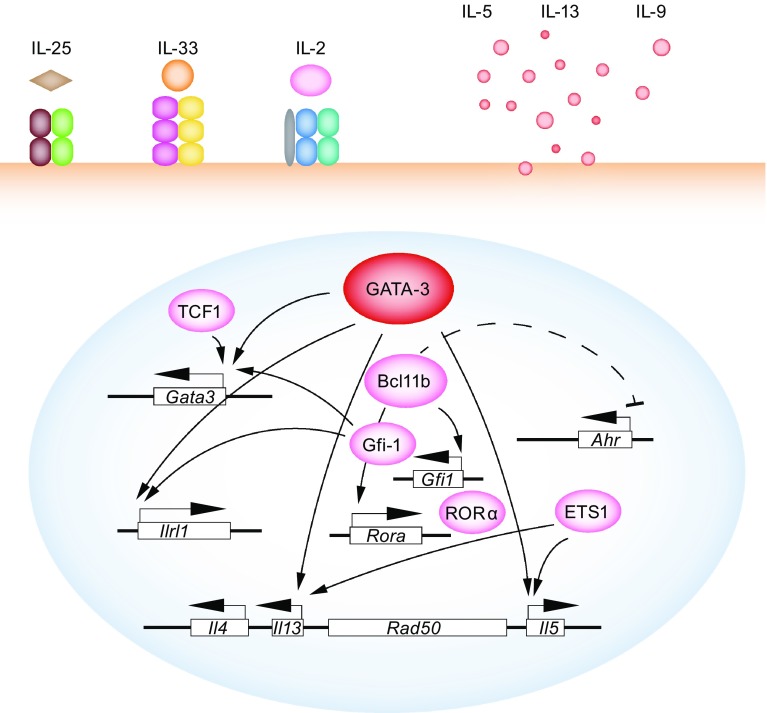
**ILC2-specific transcription factors**. GATA-3, which determines ILC2 development, maintenance and effector cytokines IL-5 and IL-13 production, is the master regulator of ILC2s. RORα, Bcl11b, ETS1, TCF1, and Gfi-1 are involved either in ILC2 development at early stages or in functional regulation of ILC2s after maturation. TCF1 regulates *Gata3* expression at a very early stage to regulate ILC2 development. RORα and Bcl11b are also specifically required for the development of ILC2 cells. Mature ILC2s express IL-17RB, T1/ST2, and CD25 on their surface, and are responsive to stimulation by IL-25, IL-33, and/or IL-2. After fate commitment to ILC2 lineage, Bcl11b is also required for the maintenance of ILC2 identity, mainly through positive regulation of *Gfi1* and *Rora* expression, and negative regulation of *Ahr* expression. Gfi-1 directly binds to the *Il1rl1* locus to regulate IL-33 receptor expression, which mainly affects the homeostasis of ILC2s in the skin tissue. ETS1 is also required for the fitness of ILC2 cells in certain tissues, and it regulates IL-5 and IL-13 production by ILC2s

ILC2s are characterized by very high expression levels of the Th2 master regulator GATA-3, which endows ILC2s with the ability to produce the Th2 effector cytokines, IL-5 and IL-13, stimulated by upstream cytokines including IL-33, IL-25, and IL-2 (Hoyler et al., 2012) (Fig. 2). GATA-3 is required not only for the generation of common ILC progenitors, but also for the maintenance and function of ILC2s. Deletion of *Gata3* in committed ILC2 cells leads to diminished cytokine production and eventually cell death (Yagi et al., 2014). GATA-3 directly binds to Th2 cytokine loci in ILC2s as in Th2 cells (Zhong et al., 2016). However, the detailed mechanism of how GATA-3 maintains mature ILC2s is still unclear. It is possible that GATA-3 may regulate cell cycle or cell apoptosis related genes in ILC2s.

Besides the master regulator GATA-3, other transcription factors such as Bcl11b, RORα, TCF1, and Gfi-1, etc. specifically affect ILC2 cell development and/or function although these factors may also be expressed by other ILCs (Fig. 2).

#### Bcl11b

B-cell CLL/lymphoma 11b (Bcl11b) is another critical transcription factor for T cell development. It promotes the transition from double negative (DN) 2a stage to DN2b stage, after which the T cell lineage fate is fully determined (Ikawa et al., 2010). The role of Bcl11b in ILC development, however, is restricted to the ILC2 subset. A subgroup of ChILP cells has been found to express Bcl11b and they appear to be the progenitors of ILC2s (Walker et al., 2015; Yu et al., 2015; Zhong and Zhu, 2015a). In *Bcl11b* ^−/−^ mice, ILC2 cells fail to develop. Mature ILC2s in various tissues also express high levels of Bcl11b while the immature ILC2s that are found in the bone marrow express negligible levels of Bcl11b. *Bcl11b* deletion in mature ILC2 cells does not affect cell survival but results in a loss of ILC2 identity (Califano et al., 2015). Some *Bcl11b*-deleted ILC2 cells up-regulate RORγt expression and may therefore acquire some ILC3 features. The conversion of ILC2s to ILC3s may be attributable to the regulation of *Gfi-1*, *RORα*, and *AhR* by Bcl11b in ILC2s (Fig. 2). Besides ILC2s, some NKp46^+^ ILC3 and ILC1 cells also express Bcl11b, however the function of Bcl11b in non-ILC2s is still elusive.

#### RORα

RORα is expressed in the progenitors as early as at the ChILP stage, however it is specifically required for the generation of ILC2s (Halim et al., 2012; Wong et al., 2012). The underlying mechanism of RORα-mediated ILC2 development is still unclear. RORα is continuously expressed in all mature ILC subsets. Although it is not clear whether RORα has any function in mature ILC1 or ILC2 cells, a recent report indicates that RORα may regulate ILC3 cells (Lo et al., 2016).

#### TCF1

Although TCF1 is expressed before Id2 during ILC development, its expression is maintained even after the ChILP stage, which is consistent with its critical function in the generation of ILC2 cells through both GATA-3-dependent and -independent mechanisms. The overexpression of TCF1 partially bypasses Notch signaling, which is required for the development of ILC2s, indicating that TCF1 mediates Notch-dependent ILC2 development (Yang et al., 2013). TCF1 is also required for the proper generation of NKp46^+^ ILC3s (Mielke et al., 2013a).

#### Gfi-1

Gfi-1 has an important regulatory function in both Th2 cells (Zhu et al., 2002; Zhu et al., 2006) and ILC2s. It has been reported that, in ILC2 cells, Gfi-1 regulates the expression of *Gata3* and *Il1rl1* (the gene encoding the IL-33 receptor) (Spooner et al., 2013) (Fig. 2). Loss of *Gfi-1* in ILC2s results in reduced GATA-3 expression but enhances RORγt expression. Such dys-regulation leads to the co-expression of ILC2 effector cytokine IL-13 and the ILC3 effector cytokine IL-17.

#### ETS1

ETS1 is a transcription factor that has been recently identified to be particularly important for ILC2 development and function (Zook et al., 2016). *Ets1* deficient mice show a substantial reduction of ILC2s in the bone marrow and lymph nodes. However, the total cell number of ILC2s in the lung of *Ets1* deficient mice is comparable to that of *Ets1* sufficient mice. Together with other reports (Saenz et al., 2013; Spooner et al., 2013), these results suggest that ILC2s in different tissues might develop from separate progenitors that have distinct developmental requirements—an important question which requires further investigation. Similar to its function in cNK cells, ETS1 regulates the fitness of ILC2s and the common ILC progenitors mainly by promoting optimal expression of Id2. In mature ILC2s, ETS1 regulates ILC2 cell proliferation and the effector cytokine IL-5 and IL-13 production in response to IL-33 or IL-25 stimulation (Fig. 2).

### ILC3-associated transcription factors

**Figure 3:**
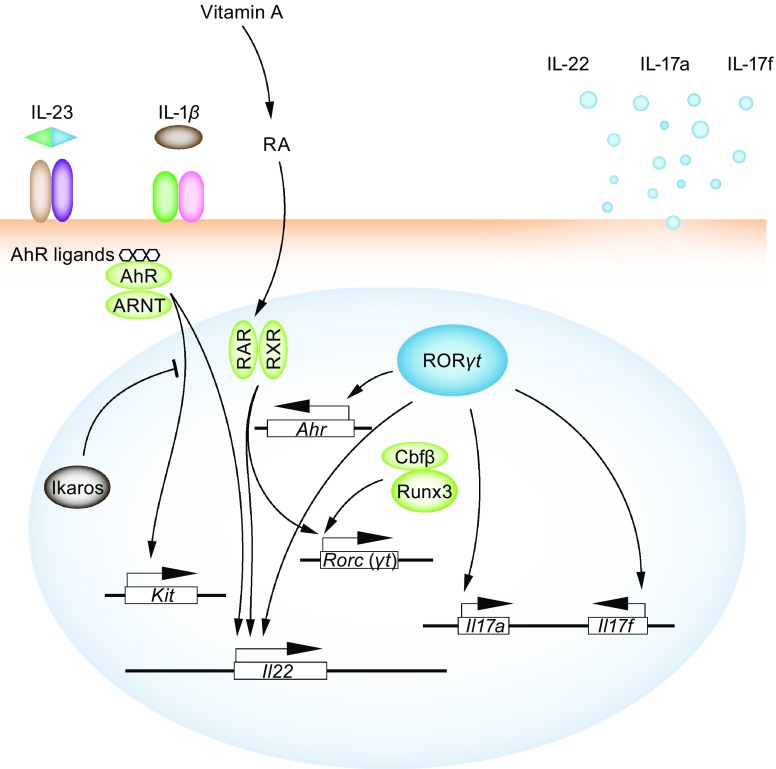
**ILC3-specific transcription factors**. RORγt acts as a master regulator for all ILC3 cells, including the CCR6^+^ LTi/LTi-like cells and the NKp46^+^ ILC3s. RORγt is also responsible for the production of ILC3 effector cytokines, such as IL-22, IL-17a, and IL-17f. AhR and RAR/RXR are expressed in ILC3 cells and sense the nutrient derived metabolites. AhR is downstream of RORγt, while RAR/RXR can promote RORγt expression and LTi cell generation in fetal stage. Upon binding to AhR ligands or retinoic acid (RA), these transcription factors can dimerize with co-activators and translocate into nucleus to regulate ILC3-specific genes including *Kit* and *Il22*, etc. Runx3 and its obligatory partner Cbfβ are also capable of regulating RORγt expression and ILC3 early development. Ikaros is a positive regulator for LTi cell generation in the fetal stage. However, in mature ILC3s in adult mice, Ikaros negatively regulates ILC3s through inhibiting the AhR signals

Like Th17 cells, RORγt-expressing ILC3s are similarly not a homogeneous population. They can be divided into at least two lineages, an LTi lineage and a natural cytotoxic receptor (NCR)-expressing ILC3 lineage (Klose et al., 2013). RORγt, as the master regulator for ILC3s, is required for the development and the maintenance of both lineages. However, the development of NCR^+^ ILC3s requires additional regulators such as T-bet and GATA-3. IL-23 and IL-1β receptors expressed in both ILC3 lineages, and can transduce the upstream cytokine signals for the production of ILC3 cytokines IL-22, IL17a, and IL-17f (Fig. 3).

#### GATA-3 and T-bet

Data from our ILC3-specific conditional *Gata3* knockout mouse indicates that GATA-3 acts upstream of T-bet to direct NCR^+^ ILC3 development from a CCR6^−^T-bet^−^ precursor (Zhong et al., 2016). GATA-3 inhibits RORγt expression by directly binding to the *Rorc* locus. Therefore, GATA-3 regulates the balance between RORγt and T-bet, which is critical for the development of NCR^+^ ILC3s. A two-fold increase or decrease in RORγt expression may dramatically change the outcome in the development of T-bet-expressing ILC3s. Once T-bet expression reaches a sufficient level, it acts as a repressor of RORγt and further promotes the generation of NCR^+^ ILC3. Besides their critical role during the development in NCR^+^ ILC3, both GATA-3 and T-bet regulate the production of ILC3 effector cytokine IL-22 (Sciume et al., 2012; Zhong et al., 2016).

#### AhR

Aryl hydrocarbon receptor (AhR), the master regulator in xenobiotic metabolism, has been reported to influence several other cellular functions including the development of immune cells (Zhou, 2016). The AhR protein contains a bHLH DNA binding domain and a Per-Arnt-Sim (PAS) domain for agonist binding. Before binding its agonist, AhR forms an inactive complex with heat shock protein 90 (Hsp90), aryl hydrocarbon receptor interacting protein (AIP) and p23 in the cytosol. After binding to its agonists, a conformational change in AhR releases itself from the complex and thus enables its translocation to the nucleus. After AhR dimerizes with another bHLH-PAS protein aryl hydrocarbon receptor nuclear translocator (ARNT), AhR initiates the transcription of its downstream target genes such as *Il22* and *Kit* in ILC3s (Fig. 3). AhR is a regulator for both LTi and NCR^+^ ILC3s. *AhR* ^−/−^ mice have a defect in the development of LTi cells and thus lack tertiary lymphoid structure cryptopatches (CPs) and isolated lymphoid follicles (ILFs) in the gut (Kiss et al., 2011). NCR^+^ ILC3 cells are also reduced in the *AhR* ^−/−^ mice (Qiu et al., 2012). One of the AhR regulated genes in ILC3s is c-kit, which is expressed in most of the ILC3 cells (Kiss et al., 2011). The frequency of *AhR* ^−/−^ ILC3s is still normal at early time point after birth, but the *AhR* ^−/−^ ILC3s are not able to survive consistent with reduced c-kit expression in such cells.

#### Runx3

Runx3 is a master regulator critical for CD8^+^ T cell development (Woolf et al., 2003). However Runx family members, including Runx1 and Runx3, and their common obligatory partner Cbfβ, are associated with LTi cell generation (Ebihara et al., 2015). A deficiency of these genes results in a defect in the generation of secondary lymphoid structures (Tachibana et al., 2011). However, since the development of CLP is also affected by *Runx* deletion, it is still not clear whether Runx proteins are specifically required for the development of ILCs. Based on a reporter expression study, Runx3 was found to be specifically expressed in ILC3s and ILC1s, but not in ILC2s (Ebihara et al., 2015). Early deletion of *Runx3* leads to a blockage of ILC3 development at the RORγt^−^ stage. Deletion of the Runx common obligatory partner *Cbfb* in mature NKp46^+^ ILC3s (*Cbfb* ^fl/fl^NKp46-Cre) also leads to a lack of NKp46^+^ ILC3 and ILC1 cells. Furthermore, Runx3 regulates RORγt and subsequent AhR expression in all ILC3 cells (Fig. 3).

#### Retinoic acid signals

Retinoic acid (RA) is the active metabolite of Vitamin A, which activates the nuclear receptors retinoic acid receptor (RAR) or retinoid X receptor (RXR). RAR or RXR hetero-dimerizes with co-repressors in the absence of agonist ligands. Upon binding with RA, RAR or RXR dissociates with their co-repressors, recruits co-activator proteins, and promotes downstream gene transcription (Fig. 3). The RA signal is particularly critical for the generation of LTi cells in the fetal stage, largely by controlling RORγt expression (van de Pavert et al., 2014) (Fig. 3). Maternal retinoid level is thus required for setting up immune structures in the offspring. In the adult stage, RA is also reported to be associated with the migration of ILC1 and ILC3 cells in the gut as well as the IL-22 production by ILC3s (Kim et al., 2015; Mielke et al., 2013b) (Fig. 3).

#### Ikaros

Ikaros, together with Aiolos, Helios, Eos, etc., belongs to the Ikaros zinc finger transcription factor family. Ikaros is essential for regulating hematopoiesis through affecting CLP, pro-B, and NKp generation (Yoshida et al., 2010). Ikaros is also found to be crucial for fetal LTi cells as its deficiency leads to a defect in secondary lymphoid organogenesis (Schjerven et al., 2013). However, in postnatal ILC3 cells, Ikaros may repress by inhibiting AhR activity (Li et al., 2016) (Fig. 3). A *Ikzf1* (gene encode Ikaros) deficiency can result in the expansion of ILC3s and elevated effector cytokine production. Thus, the regulatory effects of Ikaros on ILC3 development and function are stage specific.

### ILC1-associated transcription factors

**Figure 4:**
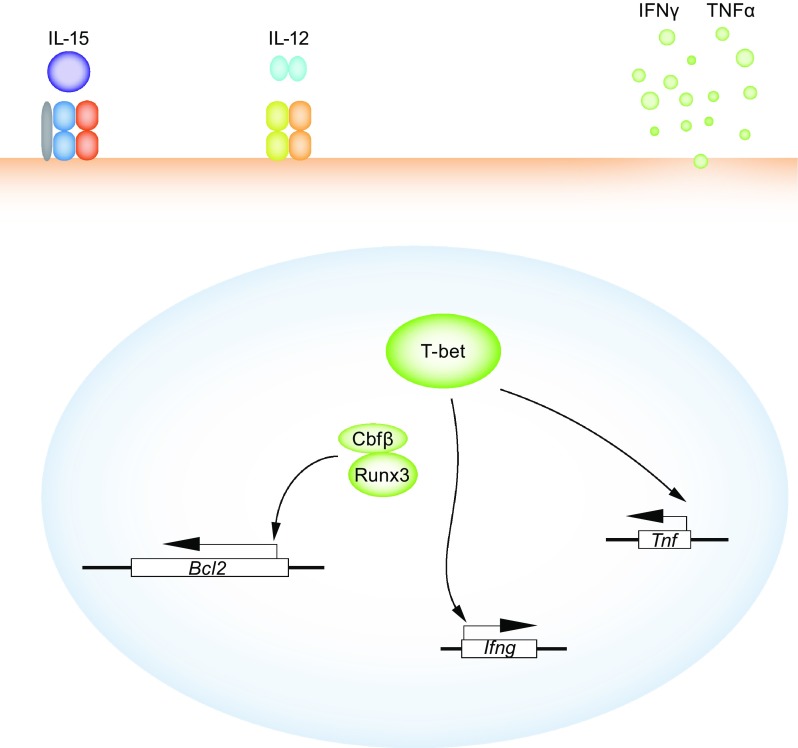
**ILC1-specific transcription factors**. ILC1 is a newly confirmed group 1 ILC population that is distinct from cNK cells. The special functions of ILC1s and underlying regulatory mechanism still need further investigation. T-bet is the master transcriptional regulator of ILC1 cells and regulates the production of ILC1 effector cytokines IFNγ and TNFα. Runx3 and Cbfβ are also required for the maintenance of mature ILC1s, mainly through regulating the expression of anti-apoptotic proteins, such as Bcl-2

ILC1 cells were identified relatively late in comparison to other ILC family members. ILC1s are much more similar to cNK cells in many of their features, however they lack cNKs cytotoxic activity. IL-15 is required for the normal development and function of ILC1s (Klose et al., 2014). IL-12, in addition, can also promote ILC1 cells to secret effector cytokines IFNγ and TNFα (Fig. 4). Based on a lineage tracing study using PLZF fate-mapping mice, ILC1s and cNK cells originate from distinct progenitors (Constantinides et al., 2015). While the vast majority of ILC1 cells develop from PLZF-expressing common ILC progenitors, most cNK progenitors do not express PLZF. ILC1 and cNK cells can be distinguished in the periphery by certain surface markers. For example, in the liver, ILC1 cells are CD49a^+^DX5^−^, whereas cNK cells are CD49a^−^DX5^+^ (Sojka et al., 2014). However, these markers are not always reliable in other tissues. Another well-accepted way to distinguish these two populations is based on their expression of Eomes (Klose et al., 2014). ILC1 cells are Eomes^−^ but cNK cells are Eomes^+^. However, it is unclear whether some ILC1s may express or have expressed Eomes during their development.

Like Th1 cells, T-bet is the master regulator for ILC1 cells. T-bet is absolutely required for the generation of ILC1 but not for cNK cells (Daussy et al., 2014), although *Tbx21* ^−/−^ cNK cells also display some abnormalities (Gordon et al., 2012). Besides its critical function during ILC1 development, T-bet is required for the maintenance of ILC1 cells and production of ILC1 effector cytokines (Klose et al., 2014) (Fig. 4).

#### Runx3

As we described above, Runx3 is essential for the development of ILC1 and ILC3, but not ILC2 cells. In committed ILC1 cells, Runx3 is also required for the survival of ILC1 cells due to its involvement in IL-15 signaling (Ebihara et al., 2015). IL-15 is particularly important for ILC1 cells as indicated by knockout studies. In mature ILC1s, the absence of *Runx3* or its common obligatory partner *Cbfb* results in enhanced apoptosis, which is associated with the dysregulation of anti-apoptotic factors such as Bcl-2 (Fig. 4).

## CONCLUSION

Transcriptional regulation underlies the functional actions of various lymphoid effectors and is the key to further understanding how the immune system works. However more comprehensive studies are still required to understand how recently identified ILC populations are generated and how their functions are controlled. Through the studies of select transcription factors, we have found that ILCs exhibit many similarities to CD4^+^ Th cells in terms of their development, maintenance, proliferation, and effector cytokine production. Beyond these similarities, the unique features of ILCs compared to those of Th cells, such as tissue residency, have recently drawn considerable attention. Despite considerable progress, there are still many challenges to address in order to understand the how ILCs and ILC-functions are ultimately regulated and controlled.
